# Band adhesions not related to previous abdominal surgery – A retrospective cohort analysis of risk factors

**DOI:** 10.1016/j.amsu.2018.11.007

**Published:** 2018-11-13

**Authors:** Andreas Skoglar, Ulf Gunnarsson, Peter Falk

**Affiliations:** a Surgical and Orthopedic Clinic, Kungälvs Hospital, Region Västra Götaland, Sweden; b Department of Surgical and Perioperative Sciences, Umeå University, Umeå, Sweden; c Department of Surgery, Institute of Clinical Sciences, Sahlgrenska Academy, University of Gothenburg, Sweden

**Keywords:** Surgical adhesion, Intestinal obstruction, Surgery-induced tissue adhesion

## Abstract

**Background:**

Postoperative intra-abdominal adhesion formation is a common cause of small bowel obstruction (SBO). Adhesions causing SBO are classed as either matted adhesions or solitary band adhesions. The aim of this study was to investigate the prevalence of previous abdominal surgery in a cohort of patients operated for bowel obstruction and to analyze the causes of obstruction discovered at surgery.

**Materials and methods:**

The study was performed at a county hospital with a catchment population of 120 000 inhabitants. Records of operations performed for bowel obstruction over a period of 70 months were retrieved.

**Results:**

*Of* the 196 surgical procedures for intestinal obstruction included, 108 (55%) were caused by adhesions. In this group, 42 (39%) were due to solitary band adhesions and 66 (61%) were due to matted adhesions. Ten of 18 male patients (56%) with a solitary obstructing band had not undergone previous abdominal surgery (p < 0.05). In the cohort as a whole, a significant number of surgical procedures were performed for solitary band adhesions in patients without prior history of surgery (p < 0.01).

**Conclusion:**

In male patients, not only previous abdominal surgery but also other factors appear to increase the risk for bowel obstruction due to a solitary band. For intestinal obstruction caused by matted adhesions, however, previous abdominal surgery is the main risk factor in both genders. Patients with signs of SBO but without previous abdominal surgery should be managed bearing in mind that solitary band adhesion and thereby strangulation may be present regardless of previous surgery or not.

## Highlights

• Post-operative abdominal adhesions are a common reason for small bowel obstruction.
• Abdominal adhesions can develop without undergoing previous surgery.
• Band adhesions occurs without prior surgery – gender seems to be of importance.

## 1 Introduction

Intra-abdominal adhesion formation after previous surgery is the most common cause of small bowel obstruction (SBO) as reported in several studies [[1], [2], [3]]. Historically, the extent of adhesions formed has been thought to depend on the magnitude of previously performed surgery. In autopsy material, adhesions have been found after minor, major and multiple abdominal operations in 51%, 72% and 93% respectively [4]. The overall incidence of adhesion-related small bowel obstruction (ASBO) after previous surgery has been estimated to be 4.6%, with the highest incidence associated with ileal pouch-anal anastomosis, followed by open colectomy (19.3% and 9.5%, respectively). For gynecologic procedures, the highest incidence occurs after open adnexal surgery [5].

Other factors contributing to adhesion formation include abrasion, desiccation and exposure to foreign materials such as gauze and talc or starch glove powder [6]. Depending on the extent of surgical trauma, it has been suggested that laparoscopic surgery reduces the tendency to form adhesions compared to open surgery [7]. Results so far published in the literature are conflicting; some authors report that open surgery quadruples the risk for SBO compared to laparoscopy [8] while other studies do not support this finding [9]. Two recently published systematic reviews did indeed find a reduction in the frequency of early and late postoperative bowel obstruction after laparoscopic compared to open procedures in colorectal surgery and appendectomy [10,11]. Postsurgical adhesions also affect later surgery, and it has been demonstrated that the duration of surgery for laparotomy increases by a median of 15 min in patients with previous surgery [12].

Adhesions are usually classed as solitary band or multiple dense matted adhesions [13]. This is of clinical importance since SBO from solitary bands is more likely to be associated with bowel ischemia and necrosis caused by strangulation compared to SBO caused by matted adhesions [14]. The nature of adhesion-related bowel obstruction seems to affect the recurrence prognosis. Readmission after surgery for SBO caused by matted adhesions has been estimated to be 49% compared to 25% for patients with a solitary band [15]. A possible gender difference in the risk for postsurgical adhesions has not been extensively studied and needs further investigation.

The purpose of this study was to explore the prevalence of previous abdominal surgery in a cohort of patients operated for bowel obstruction over a defined period of time at a county hospital, and to analyze the causes of intestinal obstruction identified at surgery.

## 2 Material and methods

### 2.1 Design

This study was performed at a regional county hospital in Sweden, with a catchment population of 120 000 according to the citizen registry. Data concerning operations for bowel obstruction over a period of 70 months, from January 1st^,^ 2006 to October 31st^,^ 2011, were retrieved from the database for perioperative planning (ORSuite^®^, IntegraSys) used by the hospital. Codes for bowel obstruction according to the International Classification of Diseases, 10th Revision (ICD-10) included: K56.5 (Intestinal adhesion with obstruction); K56.6 (Other and unspecified intestinal obstruction); K56.7 (Intestinal obstruction, unspecified); and K66.0 (Peritoneal adhesions).

The Swedish version of the Nordic Medico-Statistical Committee Classification of Surgical Procedures, version 1.9, was used to identify the type of surgical procedure performed for bowel obstruction. Search terms included: JFK00 (Division of band adhesion); JFK10 (Adhesiolysis); JFK96 (Other separation of adhesion during bowel obstruction); and JFL10 (Laparotomy and reposition or detorsion of intestine). Since miscoding has been indicated in previous papers, codes were selected to assure inclusion of all procedures related to intestinal obstruction caused by adhesions [16,17]. The medical records of all surgical procedures performed were scrutinized to assess whether the adhesions were matted or solitary band. The presence of one or several bands of adhesion tissue was classed as a solitary band adhesion while extensive diffuse adhesions were classed as matted adhesions. This work has been reported in line with the STROCSS criteria [18]. The present human cohort study was registered in accordance with the Declaration of Helsinki at www.researchregistry.com (UIN: 4171).

Demographic information was gathered from the medical records, including previous and present surgery. Extraperitoneal procedures such as inguinal or ventral hernia repair were excluded. Data were recorded on standardized case report forms and then transferred to a computer spreadsheet program (Numbers, Apple Inc. Cupertino, CA, USA).

### 2.2 Ethics

The study protocol was approved by the local Ethics Committee.

### 2.3 Statistics

The statistical tests used were based on nominal data levels, including Pearson Chi-squared test as well as uni- and bivariate logistic regression. Odds ratios (OR) are displayed with 95% confidence interval (CI: 95%). A p-value <0.05 was considered statistically significant. All statistical analyses were discussed with a statistician and were then calculated using the SPSS software (SPSS ver21, IBM, Armonk, New York, USA).

## 3 Results

**Fig. 1:**
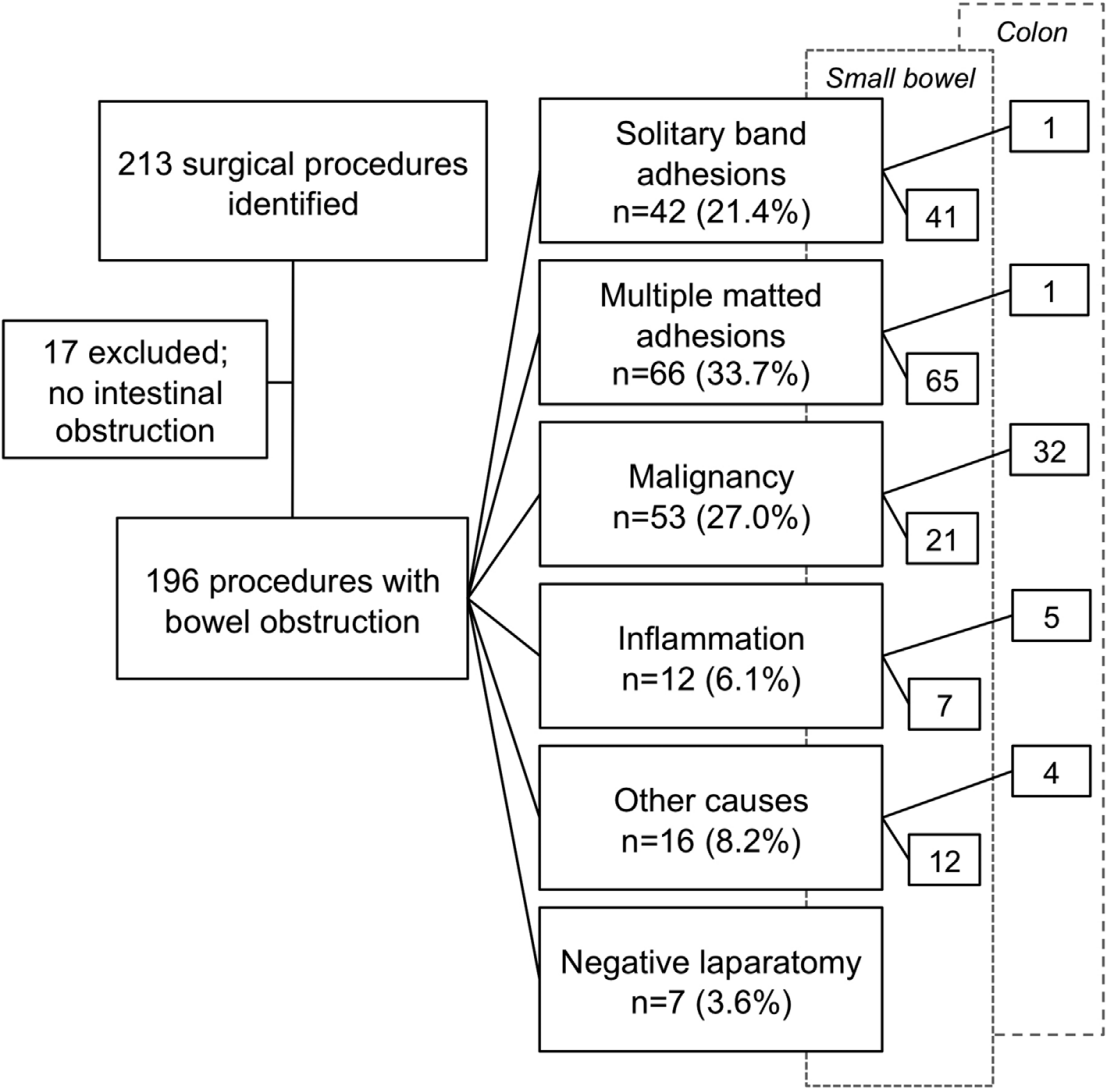
Flow chart of patients included in the study. Of 213 surgical procedures identified, 17 were excluded due to absence of bowel obstruction resulting in 196 abdominal procedures for intestinal obstruction remaining. These procedures were performed on 182 patients, (54%) women and (46%) men. Median age for women was 70 (IQR: 19 and range: 20–99) and for men 71 (IQR: 19 and range: 20–95) years.

According to the protocol, 213 operations were identified of which 17 were excluded since no bowel obstruction was found at surgery. Intestinal obstruction was evident in the remaining 196 operations, and these procedures were included in this study (Fig. 1).

The procedures included were performed on 182 patients, 99 (54%) women and 83 (46%) men (p = 0.236). Of these, 169 patients were operated once, 12 patients were operated twice and one patient was operated three times during the defined study period. The age distribution was similar between men and women with a median of 70 years for women (interquartile range [IQR] 19, range 20–99) and 71 years for men (IQR 19, range 20–95) years. In 196 procedures, 4 (2.0%) were performed on patients with a previous history of a single laparoscopic operation.

### 3.1 Bowel obstruction

The distribution of causes of bowel obstruction is shown in Table 1.

**Table 1:** Operations for bowel obstruction of all causes and previous surgery.

Cause/Previous surgery	Women (%)	Men (%)	*p*
**Obstructing solitary band adhesions**			0.003
Previous abdominal surgery	21 (87.5%)	8 (44.4%)	
No previous surgery	3 (12.5%)	10 (55.6%)	
**Total**	**24**^1^**(100%)**	**18 (100%)**	
*Previous surgery for ASBO*	*1 (4.2%)*	–	
**Matted adhesions**			0.011
Previous abdominal surgery	38 (97.4%)	21 (77.8%)	
No previous surgery	1 (2.6%)	6 (22.2%)	
**Total**	**39**^2^**(100%)**	**27 (100%)**	
*Previous surgery for ASBO*	*9 (23.1%)*	*7 (25.9%)*	
**Malignant disease**			0.893
Previous abdominal surgery	14 (51.9%)	13 (50.0%)	
No previous surgery	13 (48.1%)	13 (50.0%)	
**Total**	**27 (100%)**	**26 (100%)**	
*Previous surgery for ASBO*	*2 (7.4%)*	*2 (7.7%)*	
**Luminal obstructing inflammation**			0.558
Previous abdominal surgery	4 (66.7%)	3 (50.0%)	
No previous surgery	2 (33.3%)	3 (50.0%)	
**Total**	**6 (100%)**	**6 (100%)**	
*Previous surgery for ASBO*	*-*	*-*	
**Other causes**			0.515
Previous abdominal surgery	2 (33.3%)	5 (50.0%)	
No previous surgery	4 (66.7%)	5 (50.0%)	
**Total**	**6 (100%)**	**10 (100%)**	
*Previous surgery for ASBO*	*-*	*1 (10.0%)*	
**Negative laparatomy**			0.809
Previous abdominal surgery	1 (50.0%)	3 (60.0%)	
No previous surgery	1 (50.0%)	2 (40.0%)	
**Total**	**2 (100%)**	**5 (100%)**	
*Previous surgery for ASBO*	*-*	*-*	
**Total operations**	**104**	**92**	

1 23 operations for small bowel obstruction and one operation for large bowel obstruction.

2 38 operations for small bowel obstruction and one operation for large bowel obstruction.

Adhesion-related intestinal obstruction was found at 108 operations (55%), and all but two cases in this group had SBO. The two exceptions were: a solitary adhesive band obstructing the transverse colon in a woman with no previous history of surgery; and colon obstruction due to extensive matted adhesions in a woman with previous multiple operations for gynecologic malignancy and ASBO (Fig. 1).

Fifty-three operations (27%) were due to obstruction caused by malignant disease. The most common finding was colon obstruction caused by a tumor (32 operations), either a primary tumor of the colon or a metastasis from ovarian or prostatic cancer. The remaining 21 cases were SBO of which only three were caused by a primary neoplasm of the small intestine (carcinoid tumor in all cases). The remaining cases were caused by carcinomatosis.

Twelve cases (6%) were patients with luminal obstruction caused by inflammation. Other causes of bowel obstruction were found in 16 patients (8%) including: volvulus; gallstone; internal hernia; femoral hernia; and strictured anastomosis. These operations were erroneously registered and should have been assigned other surgical procedure codes.

The cause of intestinal obstruction was unknown in seven cases (4%), but according to the medical records these patients were suspected of having postoperative paralytic ileus, or possibly volvulus or internal hernia that had resolved spontaneously, and in one case packed feces was removed from the stoma.

### 3.2 Small bowel obstruction

In the cohort as a whole, 146 of 196 operations (74%) were due to small bowel obstruction (SBO) with no significant difference seen between genders. Adhesion was the most common cause of obstruction, resulting in 106 operations for SBO (73%) of which 87 (82% of all ASBOs) had a previous history of abdominal surgery (p < 0.001 comparing previous to no previous surgery). All causes of SBO are presented in Fig. 1.

### 3.3 Solitary bands and matted adhesions

According to the type of adhesion found at surgery, 18 men and 24 women had solitary obstructive bands whereas 27 men and 39 women had matted adhesions.

Of the 18 male patients who had a solitary band as the cause of bowel obstruction, 8 had a previous history of abdominal surgery; the remaining 10 (56%) (p < 0.05) had never had abdominal surgery. Of the 24 women with a solitary band, only 3 (13%) (p = 0.150) had never had abdominal surgery, and one patient had previously been operated for adhesion-related bowel obstruction. In all, a significant number of surgical procedures for SBO caused by solitary band adhesions were performed in patients with no previous history of surgery (p < 0.01). A summary of obstructions caused by band and matted adhesions are presented in Table 2.

**Table 2:** Risk factors for adhesion formation. Matted adhesions and band adhesions separately.

	Univariate analysis		Multivariate analysis	
	OR (95% CI)	*p*	OR (95% CI)	*p*
Matted adhesions				
Male sex	0.92 (0.42–2.02)	0.842		
Previous surgery	3.45 (1.38–8.62)	0.008	4.20 (2.22–7.98)	0.001
Previous adhesion surgery	13.1 (1.67–103.2)	0.014		
No previous surgery	0.29 (0.12–0.73)	0.008		
Solitary band adhesions				
Male sex	1.08 (0.49–2.37)	0.841		
Previous surgery	0.22 (0.07–0.67)	0.008	0.27 (0.10–0.73)	0.011
No previous surgery	3.45 (1.38–8.62)	0.008	3.78 (1.36–10.5)	0.015

A risk factor for matted adhesion was previous surgery. For band adhesion factors other than previous surgery are important.

Twenty-one of 27 men (78%) operated for obstruction caused by matted adhesions had a previous history of abdominal surgery, and seven (26%) had previously been operated for adhesion-related bowel obstruction.

This finding was even more prominent amongst the women. Of 39 operations performed on women for intestinal obstruction caused by matted adhesions, 38 patients (97%) had a previous history of abdominal surgery, and nine operations (23%) were performed on women who had had previous surgery for adhesion-related bowel obstruction. In all, 64% of procedures on men with adhesion-related bowel obstruction were performed on patients who had a previous history of abdominal surgery (P = 0.138), whereas the corresponding figure for women (including abdominal gynecologic procedures), was 94% (p < 0.001).

## 4 Discussion

Previous abdominal surgery was not found to be a dominant risk factor for small bowel obstruction caused by solitary band adhesions, unlike SBO caused by matted adhesions. Thirty-one per cent of patients (13 of 42) in whom solitary band adhesion was found to be the cause of intestinal obstruction at surgery, had never had abdominal surgery. Surprisingly, when previous abdominal surgery was dichotomized between yes or no, no previous surgery gave a significantly increased Odds Ratio in this group (p = 0.011, OR = 3.778, 95% CI: 1.361–10.485). Meissner et al. presented almost identical results where no previous history of surgery was seen in 6 of 19 (32%) cases of obstruction caused by a solitary band. However, their study was limited by the small number of procedures included [19]. These figures, however, are higher than those found in a study by Miller et al. at a single hospital between 1986 and 1996, where 17% of patients with SBO caused by a solitary band had not had previous surgery [15].

Where matted adhesion-related bowel obstruction is concerned, the picture is quite different. In the present study, 89% of patients in this category had a history of abdominal surgery, and there was no gender difference. In particular, 24% had had previous surgery for ASBO. Miller et al. found that 9% of patients conservatively and surgically treated for ASBO, had no previous history of abdominal surgery [15]. In the subgroup including patients operated for SBO caused by matted adhesions, 5% had no previous history of abdominal surgery. In the same study Miller also reported that 53% of operations for small bowel obstruction were caused by matted adhesions, 45% by a solitary adhesive band, and another 2% by a combination of both at two locations [15]. Our results are similar, with 61% of adhesive intestinal obstructions being caused by matted adhesions when other causes of intestinal obstruction such as malignancy are excluded.

In a study by Fevang et al., 83% of their patients had had one or more abdominal procedures prior to the first operation for small bowel obstruction due to adhesions [20]. This figure resembles the 81% that had a previous history of abdominal surgery in the present study when all operations for obstruction caused by adhesions are combined.

There seems to be a clear difference between solitary band adhesion-related (snare obstruction or strangulation of a loop of the small intestine) and matted adhesion-related intestinal obstruction, as regards previous history of abdominal surgery. This is of clinical importance since there is a greater risk of bowel ischemia and necrosis in SBO caused by solitary bands compared to SBO caused by matted adhesions [14]. The reason for the striking difference regarding previous history of surgery in SBO due to solitary band adhesions, between men and women, is not clear. In our study, 56% of the men who had surgery for a solitary obstructing band had no previous history of abdominal surgery, compared to 13% in women. It would seem that there are other factors, not just previous surgery, that are important in the development of solitary band adhesions. To our knowledge, this has not been reported previously. Lorentzen et al. recently found that female gender is associated with increased risk for recurrence of adhesions after surgery for ASBO [21]. These findings indicate that the impact of gender on the formation of adhesions needs further investigation.

When all operations for matted and solitary band adhesions were considered together, the difference between genders remained, with 36% of men having never had surgery prior to adhesion-related intestinal obstruction, whereas the corresponding figure for women was only 6%.

Adhesions were the cause of bowel obstruction in 55% of the operations in the present study. The overwhelming majority of these, more than 98%, were obstruction of the small intestine. Adhesions causing obstruction of the colon are uncommon but do occur, as described by Bevan [22] and Omori [23].

In the present study, we have reviewed all operations for intestinal obstruction over a defined period of time. We believe that this provides a better way of understanding causes of intestinal obstruction than exploring a cohort that includes patients treated non-surgically. Comparison with other studies is difficult due to differences in inclusion criteria. An overview of studies with inclusion criteria similar to ours is presented in Table 3 [1,15,[19], [20], [21],[24], [25], [26], [27], [28], [29], [30], [31], [32], [33], [34], [35]].

**Table 3:** Overview of previous studies with inclusion criteria similar to the present study.

Operations for small bowel obstruction (with or without previous laparotomy, all causes)										
Reference	Year	Time of study	No of operations	Solitary band	Matted adhesions	Unspecified adhesions	Tumor	Inflammation	Hernia	Other
Mucha, P Jr [23]	1987	3 years	314	–	–	49%	16%	–	15%	20%
Landercasper et al. [24]	1993	1981–1986	150	–	–	52%	11%	–	9%	29%
Franklin et al. [25]	1994	1991–1993	23	9%	35%	–	4%	–	48%	4%
Strickland et al. [26]	1999	1994–1997	40	30%	35%	–	–	3%	25%	8%
Suter et al. [27]	2000	1991–1998	83	42%	43%	–	6%	2%	2%	4%
Miller et al. [1]	2000	1986–1996	310	–	–	66%	3%	8%	4%	19%
Levard et al. [28]	2001	1988–1996	308	54%	31%	–	2%	–	5%	8%
Kirshtein et al. [29]	2005	1997–2002	65	–	–	68%	8%	2%	6%	17%
Zielinski et al. [30]	2010	2006	48	–	–	27%	35%	–	25%	13%
*Present study*	*2018*	*2006–2011*	*146*	*28%*	*45%*	*-*	*14%*	*5%*	*-*	*8%*
Operations for adhesive small bowel obstruction only (with or without previous laparotomy)										
Meissner et al. [18]	1994	1979–1993	123	15%	85%	–	–	–	–	–
Miller et al. [15] ∗	2000	1986–1996	160	45%	53%	2%	–	–	–	–
Fevang et al. [19] ∗∗	2004	1961–1995	382	63%	37%	–	–	–	–	–
Grafen et al. [31]	2010	1999–2007	93	45%	55%					
Lorentzen et al. [20]	2017	2004–2013	478	49%	51%					
*Present study*	*2018*	*2006–2011*	*106*	*39%*	*61%*	*-*	*-*	*-*	*-*	*-*
Operations for small bowel obstruction (only postoperative SBO, all cases)										
Seror et al. [32]	1993	1976–1990	80	–	–	81%	8%	–	9%	3%
Cox et al. [33]	1993	1982–1990	61	49%	33%	–	16%	–	–	2%
Nieuwenhuijzen et al. [34]	1998	1985–1994	38	–	–	74%	5%	–	8%	13%
*Present study*	*2018*	*2006–2011*	*110*	*26%*	*53%*	*-*	*13%*	*3%*	*-*	*5%*

Percentages have been rounded off and may not add to 100%.

∗ Miller et al. (2000): 160 of 204 operations included were specified with type of adhesion.

∗∗ Fevang et al. *(2004): 382 of 500 operations for first time ASBO included were specified with type of adhesion*.

During the time period of this study, a total of 6823 surgical and urological theater-based procedures were performed at the hospital. Of these 2575 were open or laparoscopic intra-abdominal procedures (hernias excluded). The 108 operations performed for small bowel obstruction caused by adhesions thus comprised 4.2% of all procedures in this study. This is a somewhat higher figure than that described by Menzies and Ellis for the period 1964–1988 at a single hospital, where 3.3% of all laparotomies were performed for adhesion-related bowel obstruction [36]. This result is surprising since we expected to find a decrease in adhesion-related bowel obstruction after the introduction of laparoscopic surgery and minimally invasive procedures. One explanation for this could be the difference in inclusion criteria between the earlier series and the present. Another possibility is the fall in the number of explorative laparotomies performed as a result of the increasing use of computer tomography, often disclosing disease not requiring surgery. Furthermore, some surgical procedures have become obsolete after the introduction of new forms of therapy. A good example of this is the rarity of ventricle resection in the management of ulcer due to the introduction of proton pump inhibitors (PPI).

Recurrence rates of adhesions after surgery for ASBO are high: Landercasper et al. estimated an adhesion-related SBO recurrence rate of 30–40% [25]; Lorentzen et al. had a recurrence rate of 12.1% after surgery for ASBO over a median follow-up of 2.2 years [21]; and Fevang et al. found cumulative recurrence rates after surgery for ASBO of 18% after 10 years and 29% at 25 years [20]. These figures are comparable to the recurrence rate in our study, and highlight the problem of recurrence after surgery for ASBO.

Beardsley et al. found adhesions to be the cause of SBO in 76% of 49 patients without a previous history of laparotomy or known disease as underlying cause [37]. In the subgroup of 34 patients requiring laparotomy, adhesions were the cause of SBO in 51%. In the present study, 36 of 146 patients with SBO had no previous history of abdominal surgery, and in 19 of those patients (53%) the cause of SBO was adhesions.

In a study by Angenete et al. the incidence of adhesion-related SBO turned out to be 0.4–13.9%. Not only previous surgery, but also age, sex and comorbidity were risk factors for adhesion-related SBO. They concluded that the risk for adhesion-related SBO after open abdominal surgery was up to 4 times the risk after a laparoscopic procedure [8].

## 5 Conclusions

In male patients, risk factors other than just previous abdominal surgery appear to play a role in adhesion-related small bowel obstruction caused by solitary band adhesion. On the other hand, previous abdominal surgery seems to be the main risk factor for future intestinal obstruction caused by matted adhesions regardless of gender. From the surgical point of view, those managing patients without a previous history of abdominal surgery who show signs of obstruction, should be aware that the risk for solitary band adhesion-related obstruction and thereby strangulation does exist.

## Conflicts of interest

The authors report no conflict of interest regarding the contents of this paper.

## Sources of funding

The project was supported by grants from the Swedish state under the agreement between the Swedish government and the county councils, the ALF-agreement (ALFGBG-143521).

## Ethical approval

The study protocol was approved by the Local Ethics Committee (University of Gothenburg, Dnr: 490–14).

## Consent

This retrospective register study was approved by the Local Ethics Committee at the University of Gothenburg (Dnr: 490–14).

This register study do not include any case reports from any patients. No names of patients, initials, volunteers or hospitals or any other details to identify individuals or specific places have been used in the manuscript.

## Author contribution

Andreas Skoglar was responsible for the idea of the paper, collecting and assembling data, searching the literature, made the tables and figures and wrote the initial part of the manuscript. Ulf Gunnarsson reviewed the tables and figures and participated in revising the manuscript. Peter Falk was responsible for the data analysis, reviewed and adjusted tables and figures and participated in the revision of the manuscript. All authors participated in the final revision and agreed to admission of this final version of the manuscript.

## Registration of research studies

The human study was registered in concordance with the declaration of Helsinki to researchregistry.com (UIN: 4171).

## Guarantor

Peter Falk is the guarantor.
